# White Matter Structural Brain Connectivity of Young Healthy Individuals With High Trait Anxiety

**DOI:** 10.3389/fneur.2019.01421

**Published:** 2020-02-13

**Authors:** Chunlan Yang, Yining Zhang, Min Lu, Jiechuan Ren, Zhimei Li

**Affiliations:** ^1^College of Life Science and Bioengineering, Beijing University of Technology, Beijing, China; ^2^Beijing Tiantan Hospital, Capital Medical University, Beijing, China

**Keywords:** high anxiety populations, diffusion tensor imaging, white matter, structural network, connectivity, young healthy individuals

## Abstract

**Background:** Although functional brain connectivity in anxiety-related disorders has been studied, brain connectivity in non-clinical populations with high trait anxiety has been rarely reported. Whether structural brain connectivity changes in young healthy individuals with high trait anxiety remains unknown.

**Methods:** Thirty-eight young healthy individuals with high anxiety levels and 34 healthy subjects with low anxiety levels who were matched by age, gender, and educational level were recruited. Diffusion tensor images were acquired to analyze white matter connectivity. A two-sample *t*-test was used for group comparison of weighted networks and graph properties.

**Results:** Different connections were detected in fractional anisotropy- and fiber number-weighted networks. These connections were widely distributed in various regions, where relative significance was located in the inter-hemispheric frontal lobe, the frontal-limbic lobe in the right intra-hemisphere, and the frontal-temporal lobe in the ipsilateral hemisphere. However, no significant difference was found in fiber length-weighted network and in graph properties among the three networks.

**Conclusions:** The structural connectivity of white matter may be a vulnerability marker. Hence, healthy individuals with high trait anxiety levels are susceptible to anxiety-related psychopathology. The findings may help elucidate the pathological mechanism of anxiety and establish interventions for populations susceptible to anxiety disorders.

## Introduction

An increasing number of young people feel large pressure caused by the fast-paced life in our modern society. The morbidity of anxiety-related disorders has increased in recent years. Trait anxiety refers to the general traits or personality and is manifested as persistent worry and instability. Anxiety is disruptive to daily life, and long-term anxiety significantly increases the risks of developing anxiety-related disorders. We supposed that young healthy individuals with high trait anxiety level may be susceptible to anxiety-related disorders (1, 2).

The analysis of brain connectivity through graph theory and magnetic resonance imaging (MRI) has become popular in research of nervous system diseases. Previous studies demonstrated abnormalities in brain connectivity among anxious patients. Zhu et al. found that changes in “small-world” properties in resting-state functional MRI (fMRI) are prominent among people with social anxiety disorders (3). Pacheco-Unguetti et al. found that trait anxiety is related to deficiencies in the executive control network, and attention-executive control function is impaired in adults with generalized anxiety disorder (4). Liao et al. found increased functional connectivity of the right posterior inferior temporal gyrus to the left inferior occipital gyrus, the right parahippocampal/hippocampal gyrus to the left middle temporal gyrus, and enhanced structural connectivity located in the genu of the corpus callosum among people with social anxiety disorders (5). Baur et al. found that the anterior insula and basolateral amygdala constitute a network markedly linked to anxiety (6).

In addition to an analysis using fMRI, electroencephalography (EEG) is used to detect abnormal connections in patients with anxiety disorders even at a rest state (7). However, T1-weighted imaging and diffusion tensor imaging (DTI) have been rarely used to detect structural brain connectivity. For the non-clinical population with high trait anxiety level, few neuroimaging studies have focused on brain connectivity. Our previous study used tract-based spatial statistics method to compare white matter differences among young healthy individuals with low and high trait anxiety levels (8). Alterations in the thalamus–cortical circuit and some emotion-related areas are commonly reported in anxiety-related disorders (8). In the present study, three weighted networks featured by fractional anisotropy (FA), fiber number (FN), and fiber length (FL) were analyzed; and group comparisons were performed using two-sample *t*-test to explore anatomical brain connectivity changes in a healthy population with high trait anxiety levels.

We expected to find important white matter structural connections related to trait anxiety in young healthy populations with high trait anxiety levels. These findings may provide supports for establishing a neuroimaging biomarker of susceptibility to anxiety disorders to help prevent and treat the disease.

## Materials and Methods

### Subjects

Seventy-two healthy right-handed undergraduate or postgraduate students were recruited from the Southwest University Longitudinal Imaging Multimodal, Brain Data Repository (China) (http://fcon_1000.projects.nitrc.org/indi/retro/southwestuni_qiu_index.html) (9). Self-rating scores including State-Trait Anxiety Inventory (10) and Combined Raven's matrix test (CRT) (11) were measured. Subjects with trait anxiety scores exceeding 50 were classified into the high trait anxiety (HTA) group (19 males and 19 females, trait score 54.5 ± 2.628), and those with scores below 30 were classified into the low trait anxiety (LTA) group (14 males and 20 females, trait score 26.177 ± 2.516) (12). The participants were matched by age (*t* = −0.567, *p* = 0.572) and gender (χ^2^ = 0.563, *p* = 0.453). None of the subjects presented a history of neurological or psychiatric disorders nor underwent mental health treatment and medications. All subjects conformed to the standards of MRI scanning and provided informed written consent prior to the study. The procedures of consent and experiments were approved by the Research Ethics Committee of the Brain Imaging Center of Southwest University and agreed with the standards of the Declaration of Helsinki (1989).

### Procedures

#### MRI Data Acquisition

All participants were scanned using a 3.0-T Siemens Trio MRI scanner (Siemens Medical, Erlangen, Germany). Diffusion-weighted imaging (DWI) for each subject was acquired with a single-shot, spin-echo, echo-planar imaging (EPI) sequence [TR/TE = 11,000/98 ms, matrix = 128 × 128, field of view (FOV) = 256 × 256 mm^2^, voxel size = 2.0 × 2.0 × 2.0 mm^3^, 60 axial slices, slice thickness = 2.0 mm, *b* value 1 = 0 s/mm^2^, *b* value 2 = 1,000 s/mm^2^] in 30 directions. The subjects were repeatedly scanned three times to increase the signal-to-noise ratio (SNR) in the DWI sequence. For each time, one *b*0 = 0 volume and 30 *b*0 = 1,000 volumes were acquired.

In addition, a magnetization-prepared rapid gradient echo (MPRAGE) sequence was used to acquire high-resolution T1-weighted anatomical images. T1-weighted structural images would be used as the templates of brain regions in the following data analysis procedure. The main parameters are as follows: repetition time = 1,900 ms, echo time = 2.52 ms, inversion time = 900 ms, flip angle = 9°, resolution matrix = 256 × 256, slices = 176, thickness = 1.0 mm, and voxel size = 1 × 1 × 1 mm^3^. In this study, T1 images were non-linearly transformed into the Montreal Neurological Institute (MNI) space, using the ICBM152 T1 template as a reference.

### Data Analysis

**Figure 1:**
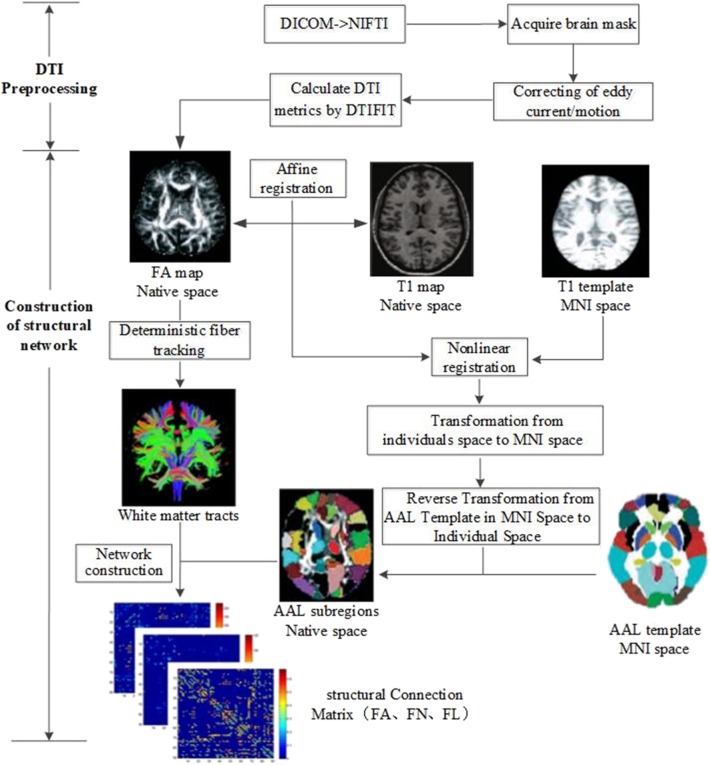
Flow chart of data analysis for image preprocessing and structural network construction.

All imaging data were processed by PANDA software (13), a MATLAB toolbox that integrates MRIcron (https://www.nitrc.org/projects/mricron), FSL (https://fsl.fmrib.ox.ac.uk/fsl/fslwiki), and Diffusion Toolkit (http://www.trackvis.org/dtk/). The flowchart of data analysis is shown in Figure 1. The procedure using PANDA mainly comprises data preprocessing and construction of white matter structural network.

#### Image Preprocessing

In detail, the following data preprocessing steps were performed: (1) checking of image quality and converting of DICOM files into NIFTI images; (2) brain extraction and brain mask estimation; (3) cropping of images and correcting of eddy current/motion; and (4) calculation of DTI metrics (FA).

#### Construction of White Matter Structural Networks

After preprocessing, white matter structural networks were constructed. (1) Affine transformation was used to match FA map and its corresponding T1 image in individual space. (2) The transformed T1 map was non-linearly registered into MNI space by T1 template. (3) Based on the above two steps, non-linear transformation from DTI individual space to MNI standard space and its inverse transformation were obtained. The Automated Anatomical Labeling (AAL) template (14) including 90 brain subregions in MNI space was registered into individual space by inverse transformation. Ninety subregions in the individual space were then segmented and labeled. (4) White matter fibers were tracked with deterministic tracking algorithm. When FA <0.2 or direction change > 45°, the white matter fiber tracking was terminated to acquire the whole brain tractography. (5) Based on the whole brain tractography, white matter structural networks could be constructed by defining FA, FN, and FL as edge weight and 90 subregions as nodes.

### Network Analysis

Graph theoretical network analysis was performed to investigate potential differences in structural network topological characteristics between HTA and LTA groups. Edges with FN value <3 and FA <0.2 were excluded to eliminate the pseudoconnection or noise of the network (15). Accordingly, FA-weighted, FN-weighted, and FL-weighted matrix for the following analysis were obtained. The metrics of these networks such as global efficiency (Eg), local efficiency (Eloc), clustering coefficient (Cp), characteristic path length (Lp), and small-world property (Sigma) were calculated by GRETNA software (16). Global efficiency refers to the average inverse shortest path length, which reflects the overall efficiency of information transmission in the network. Clustering coefficient was described as the prevalence of clustered connectivity around individual nodes. The average shortest path length between all pairs of nodes in the network was called the characteristic path length (17). Local efficiency was defined as the average efficiency of the subnetwork, which described the information exchange efficiency of subnetworks. Small-world property indicated a network with high global efficiency and local efficiency (18).

In addition, edges of the three white matter connectivity matrices (FA, FN, and FL) between HTA and LTA groups were compared to identify alterations in fiber connection. The different connections were displayed using Brainnet viewer software (19).

### Statistical Analysis

Differences in structural network properties (Sigma, Eg, Eloc, Cp, and Lp) and white matter connectivities between HTA and LTA groups were assessed by two-sample *t*-test. The false discovery rate (FDR) correction for multiple comparisons was used. In addition, given that depression may influence the results, the Beck Depression Inventory (BDI) score was regarded as a covariate. The above analysis was performed by GRETNA software.

## Results

### Demographic Characteristics and Behavioral Data

Demographic characteristics and behavioral data of the subjects were compared (Table 1). No significant differences in age, gender, and CRT were observed between HTA and LTA groups.

**Table 1:** Statistics of the demographic and behavioral data (mean and SD).

**Characteristics**	**HTA**	**LTA**	**Test statistic**	***p*-value**
Gender (male/female)	38 (19/19)	34 (14/20)	χ^2^ = 0.563	0.453
Age (years)	20.237 ± 1.384	20.471 ± 2.078	*t* = −0.567	0.572
TAI	54.50 ± 2.628	26.177 ± 2.516	*t* = 46.58	<0.001^*^
SAI	43.026 ± 9.356	24.882 ± 3.883	*t* = 10.947	<0.001^*^
BDI	13.421 ± 7.366	2.294 ± 2.877	*t* = 8.608	<0.001^*^
CRT	66.263 ± 4.157	66.088 ± 3.108	*t* = 0.204	0.839

*SD, standard deviation; HTA, high trait anxiety; LTA, low trait anxiety; TAI, trait anxiety inventory; SAI, State Anxiety Inventory; BDI, Beck's Depression Inventory; CRT, combined Raven's matrix test*.

* *p < 0.001*.

### Alterations in Structural Networks

The topological properties of the three structural networks were not significantly different. However, some altered connections were detected in FA- and FN-weighted networks.

#### Fractional Anisotropy-Weighted Network

**Figure 2:**
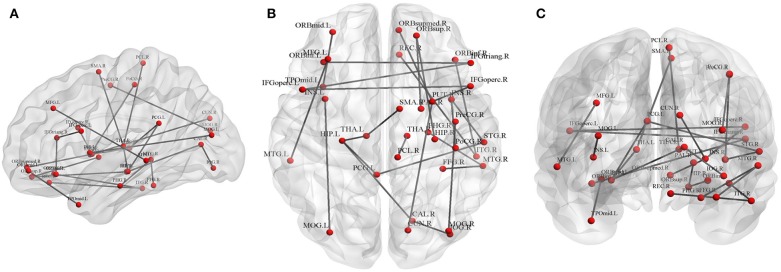
Graphical representation of different connections in fractional anisotropy (FA)-weighted network. **(A)** Sagittal plane, **(B)** axial plane, and **(C)** coronal plane.

Twenty-five connections featured by FA value decreased significantly in the HTA group compared with the LTA group (Table 2 and Figure 2) (*p* < 0.01, FDR corrected). The most different connections primarily comprised the connectivity of the left inferior orbitofrontal gyrus to the right inferior frontal gyrus (triangular), the right superior orbitofrontal gyrus to the right hippocampus, the right medial orbitofrontal gyrus to the right lenticular nucleus (putamen), the left hippocampus to the left posterior cingulate gyrus, the left thalamus, the right calcarine, the right superior temporal gyrus to the left posterior cingulate gyrus, and the right middle occipital gyrus to the right precentral gyrus (*p* < 0.001, FDR corrected).

**Table 2:** Different connections in FA-weighted network.

**Different connections**		***p*-value**
**Frontal lobe–frontal lobe**		
Inferior frontal gyrus (opercular)_L	Inferior frontal gyrus (opercular)_R	0.001866^*^
Inferior frontal gyrus (opercular)_L	Inferior frontal gyrus (triangular)_R	0.001003^*^
Inferior orbitofrontal gyrus_L	Inferior frontal gyrus (triangular)_R	0.000043^**^
**Frontal lobe–limbic lobe**		
Superior orbitofrontal gyrus_R	Hippocampus_R	0.000768^**^
Superior orbitofrontal gyrus_R	Lenticular nucleus, pallidum_R	0.002447^*^
Inferior frontal gyrus (opercular)_R	Lenticular nucleus, putamen_R	0.007135^*^
Supplementary motor area_R	Thalamus_L	0.008487^*^
Medial orbitofrontal gyrus_R	Lenticular nucleus, putamen_R	0.000383^**^
Paracentral lobule_R	Thalamus_R	0.006736^*^
**Frontal lobe–temporal lobe**		
Inferior orbitofrontal gyrus _R	Inferior temporal gyrus_R	0.002246^*^
Middle frontal gyrus_L	Middle temporal gyrus_L	0.007896^*^
Middle orbitofrontal gyrus_L	Temporal_Pole_Mid_L	0.009882^*^
Rectus gyrus_R	Inferior temporal gyrus_R	0.005715^*^
**Hippocampus–brain regions**		
Hippocampus_L	Posterior cingulate gyrus_L	0.000014^**^
Hippocampus_L	Thalamus_L	0.000824^**^
Hippocampus_L	Calcarine_R	0.000043^**^
ParaHippocampal gyrus_R	Middle temporal gyrus_R	0.002421^*^
**Temporal lobe–brain regions**		
Superior temporal gyrus_R	Posterior cingulate gyrus_L	0.000043^**^
Fusiform gyrus_R	Middle temporal gyrus_R	0.003185^*^
**Occipital lobe–brain regions**		
Middle occipital gyrus_R	Precentral gyrus_R	0.000436^**^
Cuneus_R	Lenticular nucleus, putamen_R	0.007813^*^
Calcarine_R	Inferior occipital gyrus_R	0.003976^*^
**Insula–brain regions**		
Insula_L	Middle occipital gyrus_L	0.009393^*^
Insula_R	Postcentral gyrus_R	0.005002^*^
Insula_R	Superior temporal gyrus_R	0.005260^*^

*FA, fractional anisotropy; FDR, false discovery rate; L, left hemisphere; R, right hemisphere*.

* *p < 0.01*,

** *p < 0.001; FDR corrected*.

#### Fiber Number-Weighted Network

**Figure 3:**
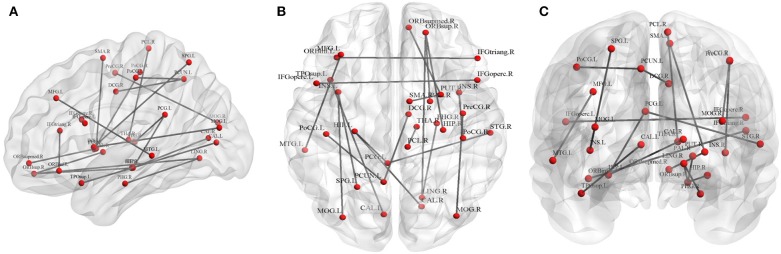
Graphical representation of different connections in fiber number (FN)-weighted network. **(A)** Sagittal plane, **(B)** axial plane, and **(C)** coronal plane.

Twenty connections were detected to have differences (*p* < 0.01, FDR corrected, Table 3 and Figure 3). The most different connections primarily comprised the connectivity of the left inferior frontal gyrus (opercular) to the right inferior frontal gyrus (opercular), the right inferior frontal gyrus (triangular) to the left inferior orbitofrontal gyrus, the right superior orbitofrontal gyrus to the right hippocampus, the left hippocampus to the right calcarine and the left posterior cingulate gyrus, the right middle occipital gyrus to the right precentral gyrus, and the right superior temporal gyrus to the left posterior cingulate gyrus (*p* < 0.001, FDR corrected).

**Table 3:** Different connections in FN-weighted network.

**Different connections**		***p*-value**
**Frontal lobe–frontal lobe**		
Inferior frontal gyrus (opercular)_L	Inferior frontal gyrus (opercular)_R	0.000009^**^
Inferior frontal gyrus (triangular)_R	Inferior orbitofrontal gyrus _L	0.000043^**^
**Frontal lobe–limbic lobe**		
Superior orbitofrontal gyrus_R	Hippocampus_R	0.000025^**^
Superior orbitofrontal gyrus_R	Lenticular nucleus, pallidum_R	0.006374^*^
Paracentral lobule_R	Thalamus_R	0.005746^*^
Supplementary motor area_R	Lenticular nucleus, putamen_R	0.001449^*^
Medial orbitofrontal gyrus_R	Lenticular nucleus, putamen_R	0.005243^*^
**Hippocampus–brain regions**		
Hippocampus_L	Calcarine_R	0.000043^**^
ParaHippocampal gyrus_R	Lingual gyrus_R	0.004239^*^
Hippocampus_L	Posterior cingulate gyrus_L	0.000092^**^
Middle cingulate gyrus_R	Precuneus_L	0.006184^*^
**Insula–brain regions**		
Insula_L	Superior parietal gyrus_L	0.009418^*^
Insula_R	Postcentral gyrus_R	0.003178^*^
Insula_L	Middle occipital gyrus_L	0.003504^*^
**Occipital lobe–brain regions**		
Calcarine_L	Temporal pole:Superior temporal pole_L	0.009510^*^
Lingual gyrus_R	Superior orbitofrontal gyrus_R	0.006624^*^
Middle occipital gyrus_R	Precentral gyrus_R	0.000493^**^
Precuneus_L	Postcentral gyrus_L	0.002417^*^
**Temporal lobe–brain regions**		
Middle temporal gyrus_L	Middle frontal gyrus_L	0.008624^*^
Superior temporal gyrus_R	Posterior cingulate gyrus_L	0.000043^**^

*FN, fiber number; FDR, false discovery rate; L, left hemisphere; R, right hemisphere*.

* *p < 0.01*,

** *p < 0.001, FDR corrected*.

## Discussion

To our knowledge, the investigation on white matter structural connectivity in young healthy people with high trait anxiety is limited. Our group previously used tract-based spatial statistics method to detect the changes of white matter in young healthy populations with high trait anxiety (8). In this study, our results showed widespread abnormal connections in brain regions, but no significant difference in the network property was detected. Alterations in white matter structural connectivity were primarily located in the inter-hemispheric frontal lobe, the frontal-limbic lobe in the right hemisphere, and the frontal-temporal lobe in the ipsilateral hemisphere.

Patients with generalized anxiety disorder exhibited decreased brain signal variability in widespread regions, including the visual network, the sensorimotor network, the fronto-parietal network, the limbic system, and the thalamus (20). It should be noted that in our study, all subjects are young healthy populations. Although the HTA group has a higher trait anxiety score, they are still healthy populations, and their brains have no significant decline yet in ability their deliver global information. It may be the primary reason why the global properties of the structural brain networks did not show alterations. The abnormal connections may only influence the local information transmission a bit or even has no significant influence. Another reason may be that they are young adults aged only 20 years, and their brains will have more plasticity during their development or maturity. At the early stage, the global network properties have no such sensitivity as the biomarker to describe the brain structural change. However, the results showed that some different connections occurred in FA and FN networks. These different connections may support to elucidate our understanding of the pathological mechanism of trait anxiety in young populations.

### Thalamus-Related Connections

In our previous voxel-wise study on highly anxious population, alterations in the anterior corona radiata (ACR)–thalamus pathway have been detected, as manifested by decreased FA values in the bilateral corona radiata and anterior thalamic radiation regions. This study also showed abnormal connectivity between the left hippocampus and the thalamus. The cortical–thalamus–limbic pathway is closely associated with emotional behavior and regulation related to posttraumatic stress disorder (21). Giménez et al. found that patients with social anxiety disorder showed significant functional connections between the thalamo-cortical and fronto-striatal circuits with task induction (22). These findings suggest that thalamus-related connections may be a vulnerable marker in young healthy individuals with high anxiety.

### Temporal Lobe/Hippocampus

A large number of studies reported the importance of the hippocampus/temporal lobe for negative emotionality. Montag et al. found that four white matter tracts linking the temporal lobe/hippocampus to other brain regions were strongly correlated with trait anxiety in male participants only (23). Similarly, we demonstrated that population with high anxiety manifested significant abnormalities in 11 white matter tracts linking the temporal lobe/hippocampus to several brain regions. This study also demonstrated the abnormal connectivity of the hippocampus regions to the posterior cingulate gyrus in highly anxious populations.

### Insula

The main function of the insula is emotional processing. Baur et al. stated that the anterior insula and basolateral amygdala constitute a network that is significantly related to anxiety (6). They also supposed that the resting-state functional connection of the anterior insula to the basolateral amygdala was highly related to anxiety. Hamm et al. found that the connection of the right amygdala to the insula showed significantly increased connectivity among pediatrics with anxiety disorders (24). Yang et al. found the abnormal functional architecture of the supramarginal gyrus network and the superior parietal gyrus network in patients with anxiety disorder (25). Dennis et al. found that the enhanced connection of the left anterior insula to the default network in healthy populations with anxiety, but connections of the parahippocampal and posterior cingulate gyrus to the default network increased in adults but not in youth (26). In addition, correlations were reported between insula activity and Liebowitz Social Anxiety Scale (LSAS) (27) or Social Phobia Inventory (SPIN) (28). We also found that the connections of the insula to the occipital and parietal lobes showed abnormality in the FA- and FN-weighted networks (the left insula to the left middle occipital gyrus, the right insula to the right postcentral gyrus in FA-weighted network, the left insula to the left superior parietal gyrus, the right insula to the right postcentral gyrus, and the left insula to left middle occipital gyrus in the FN-weighted network).

### Frontal Cortex and Limbic Areas

The orbitofrontal cortex and the prefrontal cortex located in the anterior–inferior frontal lobe play a crucial role in modulation of fear *via* the amygdala (29). Baur et al. found that the volume of the left uncinate fasciculus decreased in individuals with social anxiety disorder; this finding suggests deficient structural connectivity from high-level control areas in the orbitofrontal cortex to more basal limbic areas, such as the amygdala (30). A study on functional and structural connectivity demonstrated the relationship between trait anxiety and axial diffusivity and reported a direct pathway from the anterior insula to the basolateral amygdala (6). In our study, the observed abnormalities of several frontal lobe connections comprising connectivity linking the orbitofrontal to the limbic regions may be a trait marker in white matter structural network for young healthy individuals. In the FA- and FN-weighted network, individuals with high anxiety manifested abnormalities in the three similar pathways, including the connections of the right superior frontal gyrus (orbital part) to the limbic regions (right hippocampus, pallidum, and putamen).

Basten et al. found that some regions (inferior frontal junction areas, dorsal anterior cingulate gyrus, and left fusiform gyrus) showed significantly weaker task-specific coupling for highly anxious subjects than for those with low anxiety level (31). Kim et al. found a negative functional connectivity of amygdala–dorsal to medial prefrontal cortex in subjects with high anxiety and a positive correlation with activity at rest in subjects with low anxiety (32). Hamm et al. found that anxiety disorders showed a high connection between the left amygdala and the ventromedial prefrontal cortex and posterior cingulate cortex (24). A meta-analysis of fMRI studies investigating emotional processing in individuals who excessively worry demonstrated the convergent abnormalities at the middle frontal gyrus, the inferior frontal gyrus, and the anterior insula compared with normal controls (33). During anticipation of uncertain threat, individuals with high trait anxiety level showed significantly abnormal functional activation in the thalamus, middle temporal gyrus, dorsomedial prefrontal cortex, and precuneus (34). Du et al. found that functional connectivity changed in the frontal–limbic–striatal and default-mode networks (35). In predicting uncertain threats for individuals with high trait anxiety level, it was found that the activation of the thalamus, the medial temporal gyrus, and the dorsolateral prefrontal cortex increased significantly, whereas that of the precuneus decreased (34). We also found aberrant connections in precuneus regions in high anxiety populations through an analysis of the FN-weighted network.

### Amygdala

The amygdala is a key center for processing threats and plays an important role in generation, recognition, and regulation of emotions. The amygdala is located on the medial dorsal side of the anterior temporal lobe and is a part of the limbic system. Functional brain imaging studies showed that the level of amygdala activation was higher in anxious patients than in healthy controls; moreover, increased amygdala response in clinical and healthy individuals was associated with trait anxiety (36). Makovac et al. found reduced connections of the amygdala to the prefrontal cortex in people with generalized anxiety disorders (36). Similarly, Xue et al. found that high state anxiety level was associated with decreased connectivity of the amygdala to the precuneus/posterior cingulate cortex in adults with anxiety disorders (37).

In their fMRI study, Bishop et al. found that threat-related distractors activated the rostral anterior cingulate cortex in patients with anxiety. In addition, the rostral anterior cingulate cortex and the functions of lateral–prefrontal cortex activities decreased in participants with high anxiety level (38). Similarly, the medial prefrontal cortex and anterior cingulate cortex play important roles in coping with negative emotional stimuli (emotional processing in anterior cingulate and medial prefrontal cortex). Klumpp et al. found that the bilateral anterior insula of patients with generalized social anxiety disorder exhibited strong responses to fear (39). Yang et al. found that functional connectivity among the dorsolateral prefrontal cortex, the ventral medial prefrontal cortex, and the limbic regions was enhanced in the frontal marginal circuit of emotional regulation in patients with social anxiety disorder (40). Jacob et al. found that in people with social anxiety disorders, emotion was associated with decreased connectivity between the amygdala and the prefrontal cortex (41). A task-based fMRI study concerning social anxiety disorders reported abnormal emotion processing areas (e.g., amygdala and insula) (42). However, other scholars believed that the nature of trait anxiety is related to attitudes and strategies and not to situational incentives (4). On the contrary, our results showed no abnormalities of the connections associated with the amygdala. Although the amygdala was an important brain region for anxiety disorders, it has not been structurally altered in healthy people with trait anxiety.

This study has several limitations. First, we only evaluated Chinese undergraduate students who were right-handed and had no history of mental illness. The other similar young healthy populations like those with a relatively low educational level may have results that contrast the current results. Second, the sample size of the current study is still not large enough, which may influence the precision of the results. Third, our study did not report the correlation of functional connection with our structural connection results. Nevertheless, we believe that our data provide strong support for white matter structural connectivity in young healthy individuals with high anxiety level. Results may be helpful to develop interventions before the manifestation of morbidity in young individuals with anxious disorders and to establish a microstructural biomarker of anxiety-related diseases.

## Data Availability Statement

Publicly available datasets were analyzed in this study. This data can be found here: The Southwest University Longitudinal Imaging Multimodal, Brain Data Repository (http://fcon_1000.projects.nitrc.org/indi/retro/southwestuni_qiu_index.html).

## Ethics Statement

This study was carried out in accordance with the recommendations of the Declaration of Helsinki (1989) and the Research Ethics Committee of the Brain Imaging Center of Southwest University. The protocol was approved by the Research Ethics Committee of the Brain Imaging Center of Southwest University. All subjects gave written informed consent in accordance with the Declaration of Helsinki.

## Author Contributions

CY and YZ made contributions to the conception, design, and analysis of DTI data and drafted the manuscript. ML interpreted and discussed the DTI data. JR and ZL made contributions to the revision of the final manuscript. All authors read and approved the final manuscript.

### Conflict of Interest

The authors declare that the research was conducted in the absence of any commercial or financial relationships that could be construed as a potential conflict of interest.

## Abbreviations

AAL: Automated Anatomical Labeling
ACR: anterior corona radiata
BDI: Beck Depression Inventory
CRT: combined Raven's matrix test
DTI: diffusion tensor imaging
EEG: electroencephalography
FA: fractional anisotropy
FDR: false discovery rate
FL: fiber length
fMRI: functional magnetic resonance imaging
FN: fiber number
HTA: high trait anxiety
LSAS: Liebowitz Social Anxiety Scale
LTA: low trait anxiety
MNI: Montreal Neurological Institute
MRI: magnetic resonance imaging
SPIN: Social Phobia Inventory.
